# Genome-Wide and Paternal Diversity Reveal a Recent Origin of Human Populations in North Africa

**DOI:** 10.1371/journal.pone.0080293

**Published:** 2013-11-27

**Authors:** Karima Fadhlaoui-Zid, Marc Haber, Begoña Martínez-Cruz, Pierre Zalloua, Amel Benammar Elgaaied, David Comas

**Affiliations:** 1 Institut de Biologia Evolutiva (Consejo Superior de Investigaciones Científicas-Pompeu Fabra University), Departament de Ciències Experimentals i de la Salut, Universitat Pompeu Fabra, Barcelona, Spain; 2 Laboratoire de Génétique, Immunologie et Pathologies Humaines, Faculté des Sciences de Tunis, Campus Universitaire El Manar II, Université el Manar, Tunis, Tunisia; 3 The Lebanese American University, Chouran, Beirut, Lebanon; University of Innsbruck, Austria

## Abstract

The geostrategic location of North Africa as a crossroad between three continents and as a stepping-stone outside Africa has evoked anthropological and genetic interest in this region. Numerous studies have described the genetic landscape of the human population in North Africa employing paternal, maternal, and biparental molecular markers. However, information from these markers which have different inheritance patterns has been mostly assessed independently, resulting in an incomplete description of the region. In this study, we analyze uniparental and genome-wide markers examining similarities or contrasts in the results and consequently provide a comprehensive description of the evolutionary history of North Africa populations. Our results show that both males and females in North Africa underwent a similar admixture history with slight differences in the proportions of admixture components. Consequently, genome-wide diversity show similar patterns with admixture tests suggesting North Africans are a mixture of ancestral populations related to current Africans and Eurasians with more affinity towards the out-of-Africa populations than to sub-Saharan Africans. We estimate from the paternal lineages that most North Africans emerged ∼15,000 years ago during the last glacial warming and that population splits started after the desiccation of the Sahara. Although most North Africans share a common admixture history, the Tunisian Berbers show long periods of genetic isolation and appear to have diverged from surrounding populations without subsequent mixture. On the other hand, continuous gene flow from the Middle East made Egyptians genetically closer to Eurasians than to other North Africans. We show that genetic diversity of today's North Africans mostly captures patterns from migrations post Last Glacial Maximum and therefore may be insufficient to inform on the initial population of the region during the Middle Paleolithic period.

## Introduction

The peopling of North Africa is particularly interesting for anthropologists and human population geneticists due to North Africa's strategic location at a crossroad between Europe, the Middle East and the rest of Africa. The area has been characterized by shifting patterns of human settlements with human movements constrained by the Mediterranean Sea and the Sahara Desert, which might have limited migrations into an east-west direction. However, recent studies have suggested that these barriers might have not been totally impermeable to human movements. Diverse migration and admixture processes appear to have played a pivotal role in shaping the peopling of North Africa since the Middle Paleolithic period. Archaeological data suggest that the earliest modern humans arrived to North Africa around 160,000 years ago (ya) [1]. Human settlements dated between 145,000 ya and 40,000 ya were associated with the Aterian lithic industry [2], [3], which was replaced by the Iberomaurusian culture during the Last Glacial Maximum [4]. During the Holocene, part of North Africa (mainly Eastern Maghreb) was characterized by the Capsian culture, which developed in situ in the Maghreb and experienced a Neolithic transition in their later phase [5], [6]. During the historical period, North Africa has been settled successively by diverse populations including Phoenicians, Romans, Vandals and Byzantines. By the end of the 7^th^ century C.E, Arab armies from the Arabian Peninsula arrived to North Africa spreading Islam and the Arabic language in the region. Subsequent migrations of Arab populations followed, in particular the 10^th^ century saw considerable movement of Bedouins to North Africa [7], [8].

Early genetic studies have identified an Upper Paleolithic component in current northern African populations, and suggested that the Neolithic transition occurred through cultural diffusion [9], [10]. Studies using autosomal markers such as short tandem repeats (STRs), polymorphic Alu insertions, HLA class II polymorphisms, and GM and KM allotypes have shown close genetic affinity of North Africans to Eurasian populations and found evidence of gene flow from sub-Saharan populations [11]–[24]. Recent genome-wide analysis of North Africans found substantial shared ancestry with the Middle East, and to a lesser extent sub-Saharan Africa and Europe (see Figure S1 for a geographical description of the region). An autochthonous Maghrebi ancestry that increases from east to west across northern Africa was also identified. It was suggested that this ancestry likely derive from “back-to-Africa” gene flow more than 12,000 ya [25]. In addition, it has been suggested that recent gene flow between the Middle East and North Africa was probably promoted by shared cultures after the Islamic expansion, increasing genetic similarities between North Africans and Middle Easterners [26]. Interestingly, genome-wide analysis also shows that increased genetic diversity in Southern Europe, which is higher than in other regions of the continent, is a result of recent gene flow from North Africa [27].

Analysis of uniparental markers have found two Y-chromosome lineages (E1b1b1a-M78 and E1b1b1b-M81) at high frequency in North African populations, although the origin and emergence of these lineages have been controversial, with some studies suggesting a Paleolithic component [28], while other studies pointing to a Neolithic origin [29]–[33]. E1b1b1a-M78 has probably emerged in Northeastern Africa [31] and is today widely distributed in North Africa, East Africa, and West Asia. E1b1b1b-M81 show high frequencies in Northwestern Africa and a high prevalence among Berbers. In particular, the Tuareg have 50% to 80% of their paternal lineages E1b1b1b-M81 [34], [35]. The Tuareg are seminomadic pastoralist groups that are mostly spread between Libya, Algeria, Mali, and Niger. They speak a Berber language and are believed to be the descendents of the Garamantes people of Fezzan, Libya (500 BC - 700 CE) [34]. Another common paternal lineage in North Africa is haplogroup J through its subtypes J1 and J2. J1 is found at high frequencies in the Arabic peninsula and has been previously associated with the Islamic expansion [36]. J2 is very frequent in the Levant/Anatolia/Iran region [37] and its spread in the Mediterranean is believed to have been facilitated by the maritime trading culture of the Phoenicians (1550 BC- 300 BC) [38]. In contrast to the Middle Eastern influence, studies have reported only limited contribution of sub-Saharan paternal lineages to the North African gene pool [39], [40]. Previous analyzes of mtDNA lineages in North African populations suggest significant Eurasian origins [41]–[43] with lineages dating back to Paleolithic times [41] and with recent gene flow from sub-Saharan Africa linked to slave trade [44]. mtDNA variations showed an East-West cline accompanied by a genetic discontinuity on the Libyan/Egyptian border, suggesting a differential gene flow in the Nile River Valley [45].

In this study, we complement our previous findings on the maternal lineages by analyzing Y-chromosome and genome-wide markers in North Africans. We analyze Y-chromosome markers in more than 3,000 samples from African and Eurasian populations including 302 new samples from Libya and Morocco. In addition, we explore recently published genome-wide data from North Africa, the Middle East, and Europe using new methodologies to infer on populations' relations. We ask specific questions relating to past demographic processes to reconstruct a comprehensive description of the evolutionary history of North Africa populations: 1- Do female and male lineages show similar patterns of admixture and gene flow or they have contrasting histories similar to the contrast seen in neighboring regions [46]? 2- Can we correlate diversity from uniparental markers to diversity from genome-wide SNPs? 3- North Africa has witnessed dramatic environmental changes and has also been a scene to major historical events; what is the consequence of such factors on human genetic diversity? 4- And finally, does the genetic diversity of today's North Africans reflect patterns of modern human settlement in the region during the Middle Paleolithic period?

## Materials and Methods

### Ethic statements

Written informed consent was obtained from the participants and analyses were performed anonymously. The present project (2010/3746/I) obtained the ethics approval from the local Institutional Review Board, Comitè Ètic d'Investigació Clínica – Institut Municipal d'Assistència Sanitària (CEIC-IMAS) in Spain.

### Y-chromosome Analysis

#### Subjects and Comparative Datasets

We have genotyped 302 unrelated males belonging to the general population of Libya (215) and Central Morocco (87). Genealogical information of the donors was recorded for a minimum of two generations to ascertain their paternal ancestry. All samples were procured with informed consent following the ethical guidelines specified by the Institutional Review Board of the Comitè Ètic d'Investigació Clínica-Institut Municipal d'Assistència Sanitària (CEIC-IMAS) in Barcelona, Spain.

For comparative purposes, additional published samples (2,854) from Africa, the Middle East and Europe were included in the analyses (Table S1). The YCC nomenclature [47] was used throughout the manuscript. The Tunisian populations [39] were pooled into one group since Analysis of the Molecular Variance (AMOVA) showed them to be genetically homogeneous (variation among groups  = 0.70%, p>0.05 and 1.50%, p>0.05 for Y-STR and Y-SNP, respectively).

#### Genotyping

DNA was extracted from blood samples using a standard phenol/chloroform protocol [48] and then quantified using the Quantifiler® Human DNA Quantification Kit (Applied Biosystems). Samples were genotyped with a set of fifty-five Y-chromosome SNPs in a hierarchical method using TaqMan® probes (Applied Biosystems). Real-time PCR was performed using a 7900HT Fast Real-Time PCR System (Applied Biosystems) as previously described [39].

Samples were additionally genotyped for seventeen Y-chromosome STRs using the Amp*l*STR® Yfiler® PCR Amplification Kit (Applied Biosystems) and a 3130xl Genetic Analyzer (Applied Biosystems).

#### Statistical analyses

A graphical representation (contour map) of the geographical distribution of Y-chromosome haplogroups frequencies (Table S2) was plotted using Surfer 8.0 (Golden Software Products).

The phylogenetic relationship between haplotypes belonging to E1b1b1b-M81, E1b1b1a E-M78, J1-M267 and J2-M172 haplogroups was inferred through reduced-median networks using Network 4.5.0.1 [49]. Networks were constructed using markers shared across studies: DYS19, DYS389I, DYS389b, DYS390, DYS391, DYS392, DYS393, DYS437, DYS438 and DYS439. Locus DYS389b was calculated by subtracting the DYS389I from DYS389II (co-amplified fragments).

To study the genetic diversity within populations, we calculated haplotype and haplogroup frequencies, haplogroup and haplotype diversity, and mean number of pairwise differences (MPD), using Arlequin 3.5 [50]. Non-metric multidimensional scaling (MDS) was performed in R [51] using R_ST_ distances between populations computed by Arlequin on DYS19, DYS389I, DYS389b, DYS390, DYS391, DYS392, DYS393, DYS437, DYS438, DYS439. A principal component analysis (PCA) [52] was performed on relative haplogroup frequencies normalized within populations, centered, and without variance normalization. Since haplogroup resolution was not uniform across studies, the haplogroups were reduced to the most informative derived markers shared across studies.

In order to examine the potential signals of population structure in North African populations, a hierarchical analysis of molecular variance (AMOVA) was carried out grouping the populations according to geographical criteria. The main null hypothesis tested by AMOVA was the non-differentiation of Western and Eastern North African populations. Detailed grouping designs are shown in Table S3. AMOVA analyses were performed with Y-STR haplotypes and Y-SNP haplogroups independently using Arlequin 3.5 [50].

We have used BATWING [53] to explore demographic factors such as population growth and historical splitting into sub-populations. We considered a model of exponential growth from a constant-size ancestral population. STRs used to draw the global phylogenetic tree were those used to plot the MDS. Additional four STRs (DYS448, DYS456, DYS458, GATA H4) were added to the comparisons of North Africans. STRs were assigned observed germline mutation rates [54]. All SNPs were included and contributed to resolve the phylogenetic tree; however BATWING does not use this information for posterior estimates. Priors for initial effective population size (11,000) and growth rate (1.01) that cover wide ranges of possible values were used as previously described [55], [56] along with a male generation interval of 31 years [57]. A total of 1.5 million Markov chain Monte Carlo (MCMC) samples were kept for inferences of demographic factors. A consensus tree was generated using the Fitch program from the PHYLIP package [58].

### Genome-wide Analysis

#### Comparative datasets

Samples from North Africa [25], the Middle East [26], Europe [25], and Sub-Saharan Africa [59] were merged. PLINK [60] was used for data management and quality control. Genotyping success rate was set to 99%, sex-linked and mitochondrial SNPs removed, keeping 44,000 SNPs.

#### Population structure

PCA was performed using smartpca, part of the EIGENSOFT 3.0 package [61]. A maximum likelihood tree of human populations with mixture events was plotted using *TreeMix* [62]. *TreeMix* was also used for inference of population admixture implementing three-population tests [63]. The PCA and tree were visualized using R [51].

## Results

### Paternal lineage composition in North African populations

**Figure 1:**
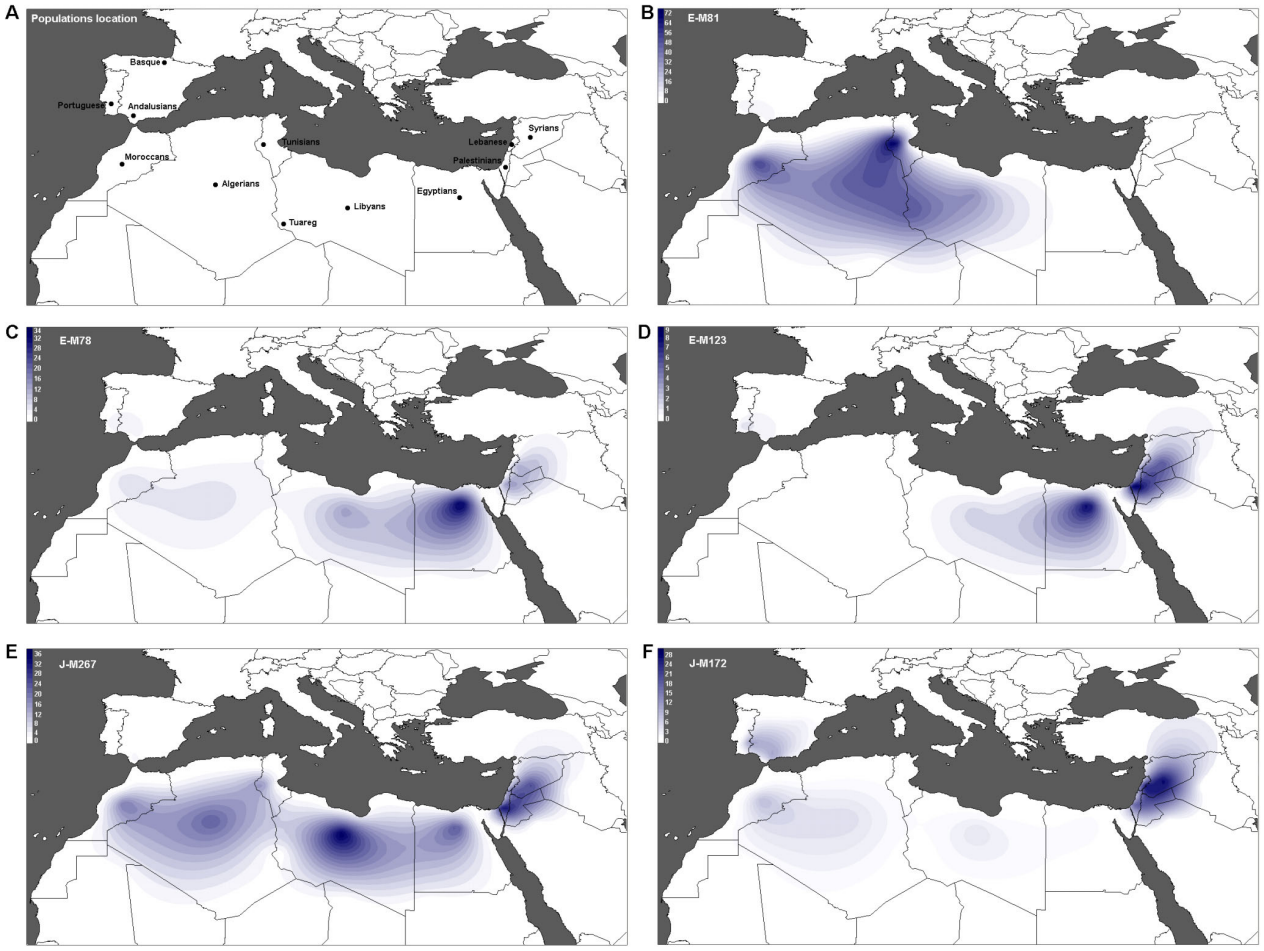
Frequency of the major Y-chromosome haplogroups in North Africa and surrounding regions. Intensity of the colors reflects the frequency of a haplogroup in the studied populations. A) Location of the analyzed populations. B–F) Frequency distribution of haplogroups E-M81, E-M78, E-M123, J-M267, and J-M172 respectively.

The paternal lineage distribution in North African populations was compared to neighboring European and Levantine groups (Figure 1A) using 302 new North African samples from Libya and Morocco (Figure S2, Table S4). As previously reported [28]–[30], [39], the two specific North African haplogroups, E1b1b1a-M78 and E1b1b1b-M81, are predominant in North African populations. The second most frequent haplogroup was J, which has been postulated to have a Middle Eastern origin [33]. Both J sub-branches, J-M267 and J-M172, were observed in North Africans. Contour maps of haplogroup frequencies show that haplogroup E-M81 is frequent in Northwest Africa but declines towards Egypt and the Levant (Figure 1B). On the other hand, E-M78 and E-M123 are frequent in the Levant and Egypt and decline towards Northwest Africa (Figure 1C and D, respectively). The Middle Eastern haplogroups J-M267 and J-M172 were observed in all samples, although with different distributions. J-M267 (Figure 1E) is prevalent in all North African and Levantine groups, whereas J-M172 is primarily distributed in the Levant and sporadically detected in North Africa and Iberia (Figure 1F).

We have studied the main haplogroups further by constructing reduced-median networks from haplotypes found in each population. The E-M81 network (Figure S3A) is characterized by a star-like shape centered on the most frequent haplotype that is present in all North African and European populations analyzed. Around 11% of the lineages clustered in specific clades within the network pointing to a high level of diversity throughout the region. The overall haplotype diversity (HD) and mean pairwise difference (MPD) values within haplogroup E-M81 are 0.8398 ± 0.0162 and 2.1693 ± 1.2055, respectively.

E-M78 network (Figure S3B) reveals high diversity within the haplogroup. This clade is mostly found in Middle Eastern populations and Northeastern Africans (27% in Libya and 33% in Egypt). Diversity values within haplogroup E-M78 are higher than for E-M81 (0.9903 ± 0.0017 and 4.1361 ± 2.0666, for HD and MPD respectively).

Network analysis of the J-M267 included 448 haplotypes, mostly from Middle Eastern populations (Figure S3D). J-M267 was found in all North Africans except the Tuareg. All North Africans also shared the modal haplotype with the Levantines. Diversity estimates within haplogroup J-M267 were 0.9524 ± 0.0067 and 2.9387 ± 1.5428 for HD and MPD, respectively.

Haplogroup J-M172 was frequent in Middle Eastern groups (73.9%), and less in Europeans (18.5%) and North Africans (7%) (Figure S3C). J-M172 network shows that clusters are shared mostly between Middle Easterners and Europeans and that most North African lineages stem out from Middle Eastern clusters.

### North African paternal population structure

**Figure 2:**
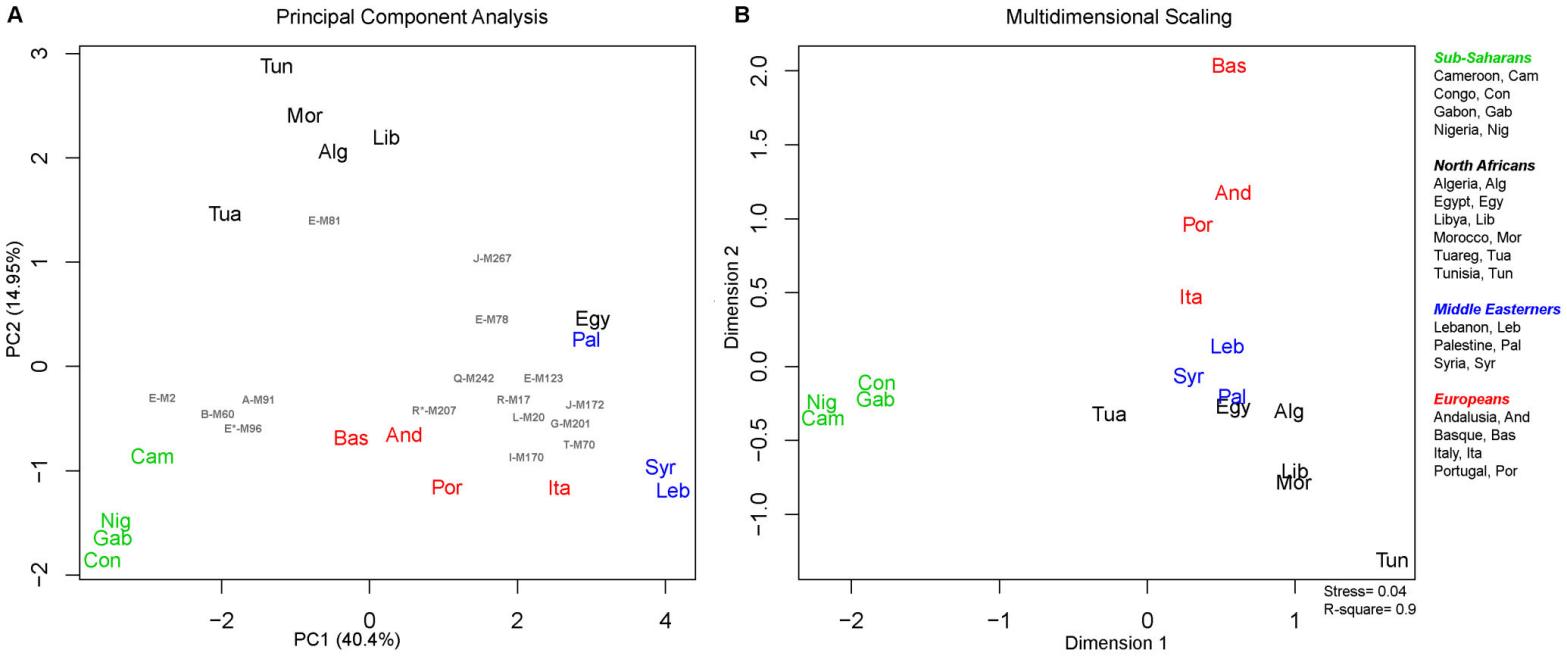
Y-chromosome population structure. A) Principal component analysis of haplogroups frequencies. B) Multidimensional scaling plot based on R_ST_ distances between populations derived from Y-STR data.

Comparison of the studied populations was first carried out using principal component analysis (PCA) on haplogroup frequencies shown in Table S2. The first two components account for 55.35% of the variation and reveal a strong geographical clustering of the populations analyzed (Figure 2A). The first component separates sub-Saharan Africans which have higher frequencies of B-M60 A-M91, E-M2, and E*-M96 haplogroups. The first component also shows clustering of the Europeans characterized by R*-M207 and I-M170 and Middle Easterners which have higher frequencies of E-M78, E-M123, J-M267, and J-M172. The second component separates all North African populations except Egyptians from all other populations and shows that E-M81 plays a major role in this structure. The Tuareg appear to be drawn towards sub-Saharans while Egyptians clustered with Middle Easterners close to Palestinians

Genetic affinity between the studied groups was further investigated by calculating pairwise genetic distances (R_ST_) using Y-STR haplotypes. The MDS (Figure 2B) shows a geographical clustering similar to the PCA. The first dimension splits the sub-Saharan Africans from all other populations. The North Africans cluster close to Middle Easterners with Tuareg drawn towards sub-Saharans and Egypt close to Palestinians.

We have further investigated the genetic structure found in North Africa by implementing AMOVA on different geographical clusters (Table S3). A significant genetic heterogeneity was found when all populations were considered as a single group (15.17% for haplogroups and 11.15% for haplotypes). For comparisons with the mtDNA results from Fadhlaoui-Zid et al [45], two groups were considered in each analysis taking into consideration current geopolitical boundaries. Results show significant variance among groups when Morocco, Algeria and Tunisia were pooled in one group and Libya, Tuareg, Egypt and the Middle East pooled in the second group. Variance among groups decreases but remains significant when Libyans and Tuareg are added to the first group. Conversely, significant differences between groups are lost when Egyptians are added to the North African group (Table S3). This result is also reflected in the PCA and MDS and shows Egypt's strong affinity to the Middle East rather than to North Africa.

**Figure 3:**
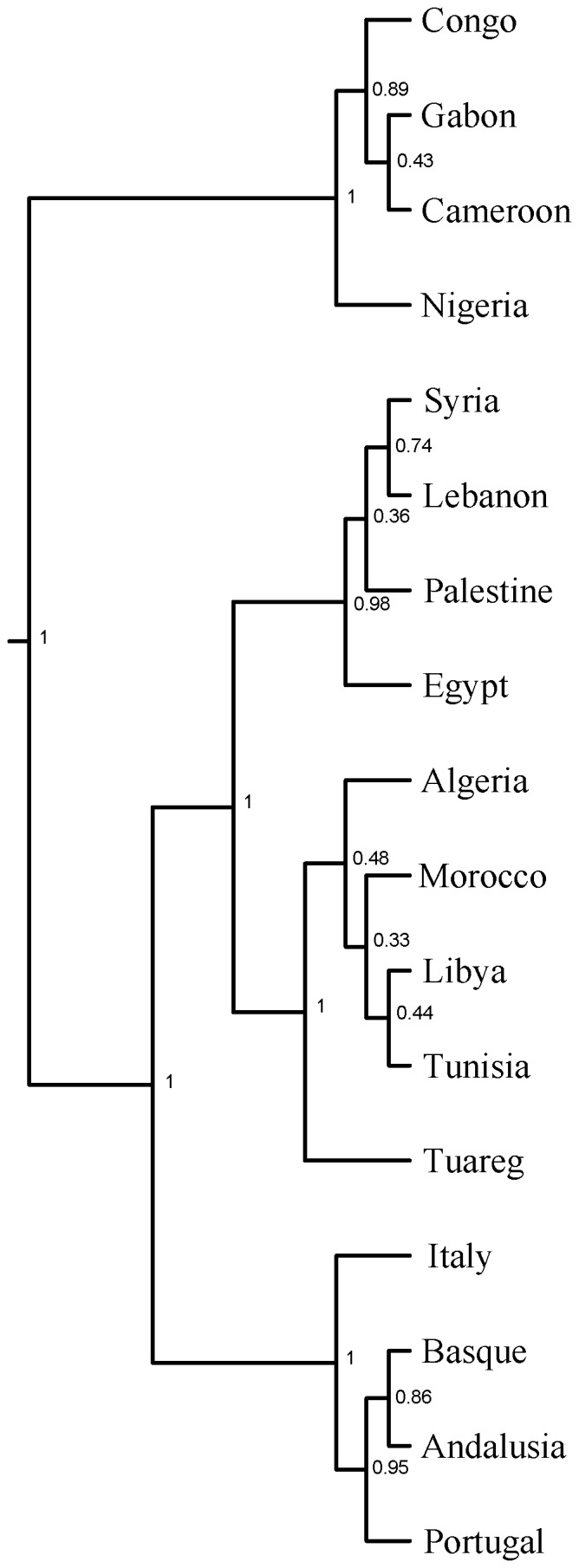
BATWING population splitting tree. Numbers on branches show partition posterior probability.

To examine population relations and the time depth in which the North African structures have emerged, we employed BATWING to create hypotheses on historical population splitting and coalescent events. BATWING results show that North Africans form their own branch, which is close to Middle Easterners (Figure 3). Egypt appears on the Middle East branch rather than with other North Africans, again in agreement with previous analyses. Our results show that most North Africans emerged around 15,000 ya during the post Last Glacial Maxima warming period (Table S5). Tunisians (Chenini-Douiret Berbers) show older dates and appear to have Paleolithic common ancestors with other North Africans. Population structure within North Africa starts with the splitting of Egypt around 2,800 ya. Tuareg split next from North Africans around 1,900 ya, followed by the remaining North Africans splitting around 1,000-1,300 ya.

### North African genome-wide population structure

**Figure 4:**
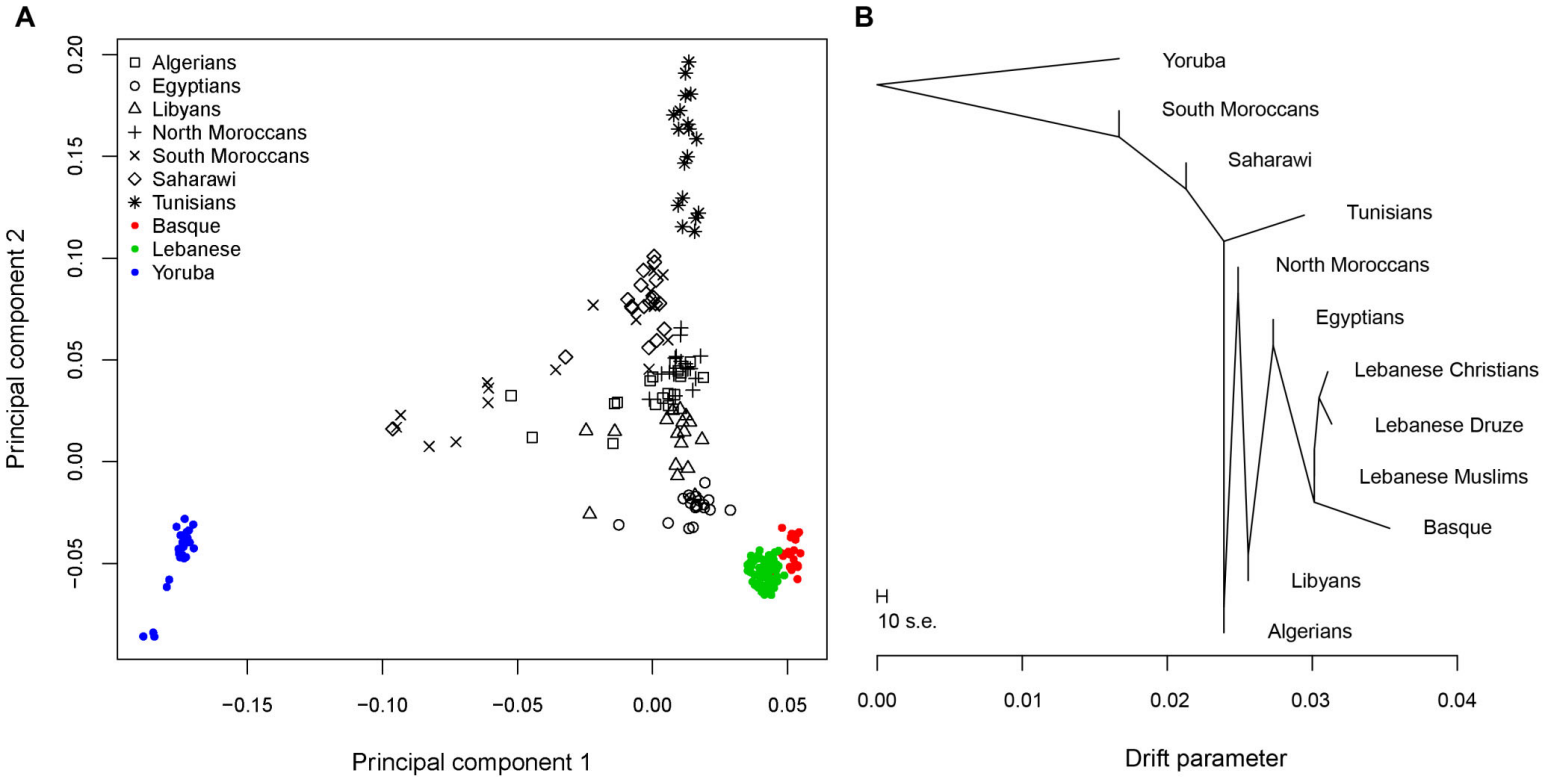
Genome-wide population structure. A) Principal component analysis of ∼44,000 SNPs showing the top two components. B) Maximum likelihood tree showing populations relationships.

PCA on genome-wide SNPs (Figure 4A) shows that North Africans are diverse and closer to Middle Easterners and Europeans than to Sub-Saharan Africans. Egyptians appear the closest to Middle Easterners and Europeans while South Moroccans are drawn towards Sub-Saharans. Tunisian samples (Chenini-Douiret Berbers) form an orthogonal cluster close but distinct from other North Africans which mostly appear in overlapping clusters.

We constructed trees that infer population relationships using *TreeMix* [62]. This method estimates both population splits and the possibility of population mixture. First, we build a maximum-likelihood tree setting the position of the root at the Yoruba (Figure 4B). South Moroccans and Saharawi appear close to Yoruba while Egyptians are on a branch leading to Middle Easterners and Basque. Next, we set *TreeMix* to allow migration edges (m) and test by increasing m sequentially up to m = 20. The initial tree structure remains mostly unchanged when migration edges are added. All North Africans except Tunisians appear admixed from an ancestral population to Yoruba. For figure clarity, we show plot m = 6 and the migration edges weights (Figure S4A). When m>6 the tree shows admixture among North Africans as well admixture with Middle Easterners/Europeans. To visually identify aspects of ancestry not captured by the tree at m = 6, we plot the residuals of the model's fit (Figure S4B). Positive residuals indicate populations where the fit might be improved by adding additional edges. *TreeMix* results show that relatedness of the tested populations cannot be explained by a simple tree; therefore we apply a 3-population test to all populations to measure treeness in the previous results. A negative value from ƒ_3_(A;B,C) implies that population A derives from at least two different groups that are related to B and C. Table S6 shows the two lowest values for each North African population. All North Africans except Tunisians appear to be a mixture of populations related to Yoruba and Eurasians (Basque and Lebanese Christians). Tunisians, Yoruba, Basque, and Lebanese Christians appear to be related to other groups by a simple tree implying a history of divergence without subsequent mixture.

## Discussion

The anthropological interest in North Africa as a crossroad between three continents and as a stepping-stone outside Africa has led to numerous studies describing the genetic landscape of the human population in this region. These studies used paternal, maternal, and biparental molecular markers to investigate population structure in North Africa. However, information from these markers which have different inheritance patterns has been mostly assessed independently, resulting in an incomplete description of North Africa populations. In this study, we analyze uniparental and genome-wide markers proved informative for inferring population origin and history. We explore our populations by examining similarities or contrasts in the results from these markers and consequently provide a thorough description of the evolutionary history of North Africa populations, trying to avoid the bias that might result by analyzing one single genomic region.

Our results from the maternally inherited mtDNA genome [45] and the paternally inherited Y-chromosome show that both males and females in North Africa underwent a similar admixture history and both are today a mixture of African and Eurasian lineages with more affinity towards the out-of-Africa populations than to sub-Saharan Africans. We should note here that although the pattern of admixture with the surrounding regions is similar in males and females, the demographic processes or historical events driving these admixtures could have been different. Also, differential sexual gene flow might have resulted in differences in the proportions of admixture components resulting in source lineage frequency differences [45]. Nevertheless, we show that a generally similar admixture history in male and female phylogenies consequently reflected on the entire genome diversity, resulting in genome-wide SNPs showing comparable patterns to uniparental markers, placing North Africans close to Eurasians. Furthermore, admixture tests using genome-wide SNPs also show that most North Africans are a mixture of populations related to current Africans and Eurasians.

Although recent cultural expansions from the Middle East, like the Islamic expansion, could have introduced new lineages to North Africa and facilitated admixture between populations from both regions, our results show that the North African component mostly formed much earlier. This is shown in the admixture tests where Basque and Lebanese Christians but not Lebanese Muslims formed potential source populations to North Africans. In particular, Lebanese Christians were shown to have been isolated for at least the last 2,000 years and were proposed to be genetically close to the ancestral population of the Levant region from which current Europeans diverged ∼15,900–9,100 ya between the last glacial warming and the start of the Neolithic [26]. Our coalescence time estimate for the paternal lineages in North Africa is ∼15,000 ya for most populations. These dates coincide with major environmental changes in North Africa following the full glacial hyperarid conditions during the Last Glacial Maxima. Humid conditions started in North Africa ∼14,500 ya transforming the area into a verdant landscape vegetated with annual grasses and shrubs which attracted hunter-gatherers who spread into the region [64]–[66]. This period was accompanied by cultural connection between the Middle East and North Africa as suggested by the lithic similarity between the regions [65].

The gradual termination of the African Humid Period started ∼6,000 ya establishing today's North Africa desert ecosystem ∼2,700 ya[65]. The desiccation of the Sahara accompanied by large-scale dust mobilization from 4,300 ya could have limited population spread and gene flow in the region, hypothetically triggering populations' divergence and structure. Our Bayesian analysis of population splits suggest North African populations started splitting ∼2,800 ya (95%CI = 1,300–4,600 ya). Egypt appears to have split first from North Africa with dates coinciding with the kingdom decline in power and conquests by Assyrians and Persians. Our results from both uniparental and autosomal markers show that today's Egyptians are genetically closer to Eurasians than to other North Africans, probably a consequence of Egypt's and the Middle East's long established interaction through conquests and trades. Tuareg split next from North Africans around 1,900 ya, followed by the remaining North Africans splitting around 1,000–1,300 ya which coincide with the Islamic expansion arriving to North Africa.

Although most North Africans appear as an admixture of populations from the surrounding regions, the Tunisian Berbers show long periods of genetic isolation, allowing a distinctive genetic component to evolve. Unlike other North Africans, our admixture tests propose that Berbers diverged from surrounding populations without subsequent mixture. We show that coalescence time estimate from paternal lineages are pushed back ∼15,000 years when Tunisians (Berbers and general population) are included in the analyses suggesting an early upper Paleolithic ancestral population with most North Africans (∼30,000–44,000 ya).

There has been recent interest in North Africa as a source for modern human migrations after most early research studying the origins of *Homo sapiens* focused on the fossils of East Africa. Recent studies of hominin fossils from northwestern Africa present strong evidence of resemblances and possible evolutionary connections with fossils representing migrations out of Africa between 130,000 and 40,000 ya [67]. Our analysis of modern North Africans shows that most populations emerged recently from admixture of Africans and Eurasians and therefore are ineffective in resolving questions about ancient human expansions. Genetic isolates, like the Tunisian Berbers analyzed here, could provide some insights on early human movements in North Africa. However, information from today's populations is limited by factors such as migration, admixture, drift, and selection pressure. We show that genetic diversity of today's North Africans mostly captures patterns from migrations post Last Glacial Maximum with no traces of genetic continuity with the first human settlers in the region. Therefore, reconstruction of modern humans' history would probably require analysis of indigenous ancient DNA from human fossils.

## Supporting Information

Figure S1 **Map of populations' location.** Map shows the geographical distribution of the analyzed populations
(TIF)

Figure S2 **Y-chromosomal phylogenetic chart.** Hierarchical phylogenetic relationships and absolute frequencies of the Y-chromosomal haplogroups observed in Libyan and Moroccan populations. Nomenclature is according to Karafet et al. (2008).
(PDF)

Figure S3 **Median joining (MJ) networks.** Plotted are MJ networks of Y-STR haplotypes within haplogroups A) E-M78, B) E-M81, C) J-M172, and D) J-M267. The circle sizes are proportional to the haplotype frequencies. The smallest area is equivalent to one individual. Branch lengths are proportional to the number of mutational steps separating two haplotypes.
(TIF)

Figure S4 **Inferred population tree with mixture events.** A) Tree of population relationships inferred by *TreeMix* allowing six migration events. Horizontal branch lengths are proportional to the amount of genetic drift that has occurred on the branch. B) Residual fit from the maximum likelihood tree. Positive residuals indicate populations where the fit might be improved by adding additional edges.
(TIF)

Table S1 **Populations selected for the Y-chromosome analyses.**
(DOC)

Table S2 **Y-chromosome haplogroup frequencies in populations selected for the present study.**
(DOC)

Table S3 **Analyses of Molecular Variance (AMOVA) in North African and Middle Eastern samples based on Y-STR haplotypes and Y-SNP haplogroups.** Acronyms are listed in Table S1.
(DOC)

Table S4 **Y-chromosome haplogroups and haplotypes in individuals from Libya and Morocco.**
(XLS)

Table S5 **BATWING results showing times of demographic factors for Y-chromosomes from North Africans.**
(DOC)

Table S6 **3-population test showing gene flow to North Africans.**
(DOC)
