# Supplementary figures and images for: Genome-Wide and Paternal Diversity Reveal a Recent Origin of Human Populations in North Africa
Source: PLoS One. 2013 Nov 27;8(11):e80293. doi: 10.1371/journal.pone.0080293 (PMC3842387; doi:10.1371/journal.pone.0080293)

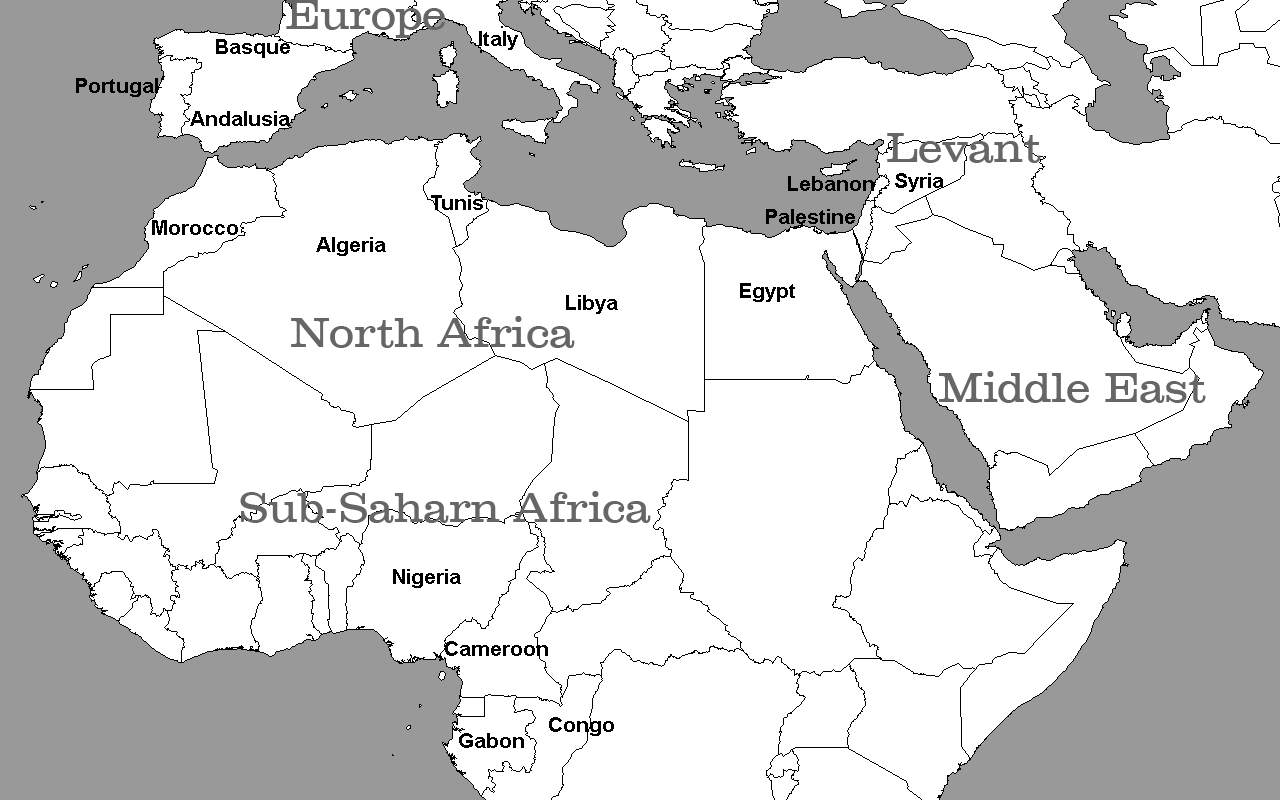

Supplement: Figure S1 — Map of populations' location. Map shows the geographical distribution of the analyzed populations (TIF) [file pone.0080293.s001.tif]

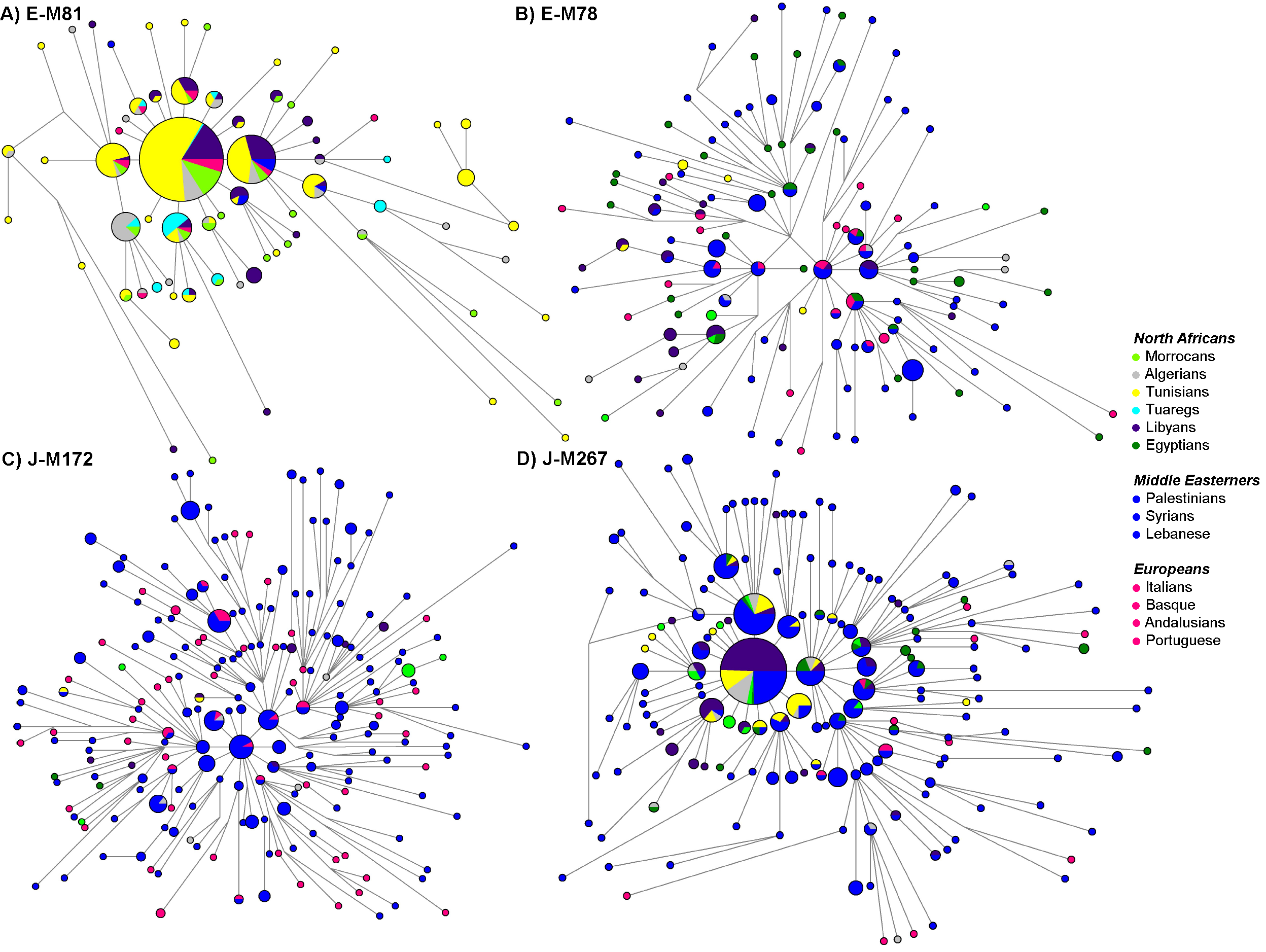

Supplement: Figure S3 — Median joining (MJ) networks. Plotted are MJ networks of Y-STR haplotypes within haplogroups A) E-M78, B) E-M81, C) J-M172, and D) J-M267. The circle sizes are proportional to the haplotype frequencies. The smallest area is equivalent to one individual. Branch lengths are proportional to the number of mutational steps separating two haplotypes. (TIF) [file pone.0080293.s003.tif]

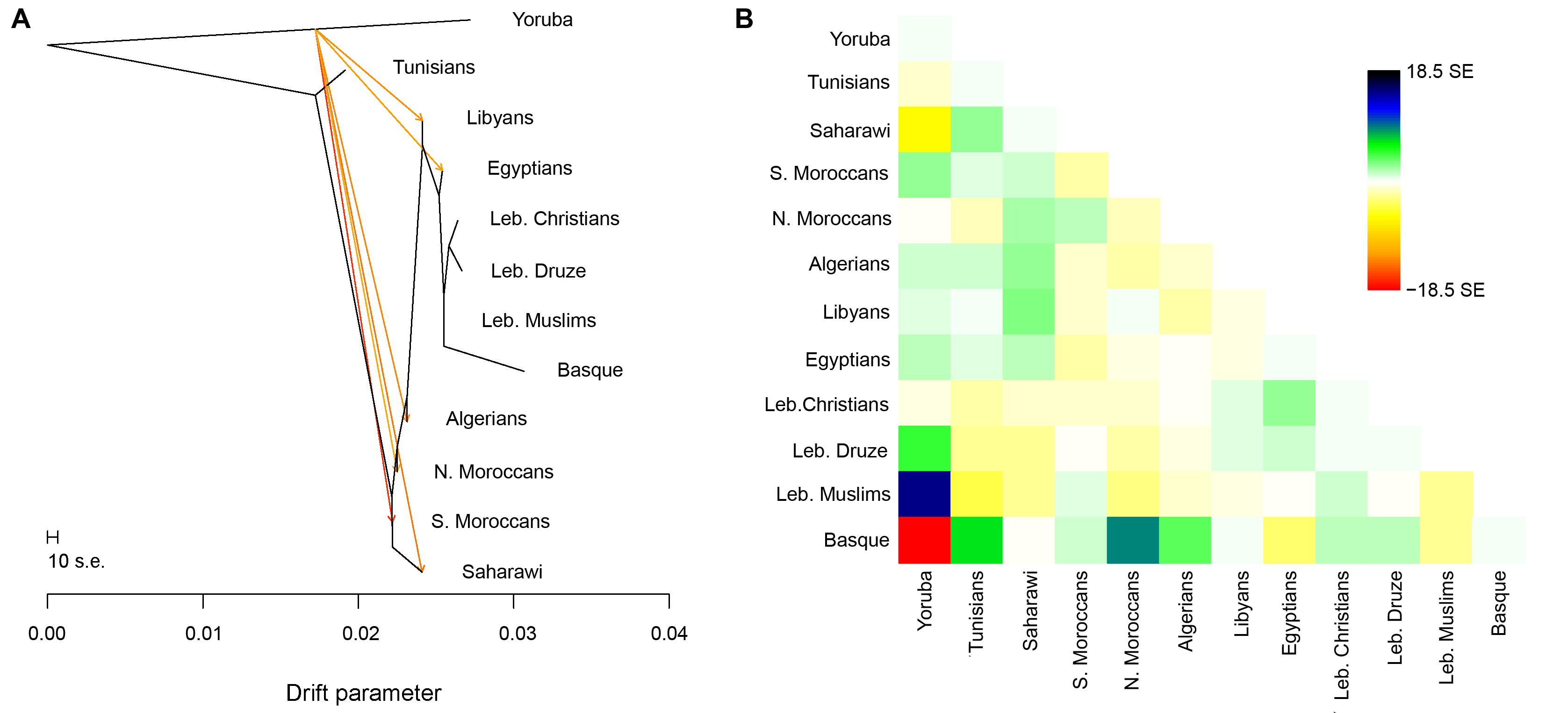

Supplement: Figure S4 — Inferred population tree with mixture events. A) Tree of population relationships inferred by TreeMix allowing six migration events. Horizontal branch lengths are proportional to the amount of genetic drift that has occurred on the branch. B) Residual fit from the maximum likelihood tree. Positive residuals indicate populations where the fit might be improved by adding additional edges. (TIF) [file pone.0080293.s004.tif]
