# Supplementary material for: Genome-Wide and Paternal Diversity Reveal a Recent Origin of Human Populations in North Africa
Source: PLoS One. 2013 Nov 27;8(11):e80293. doi: 10.1371/journal.pone.0080293 (PMC3842387; doi:10.1371/journal.pone.0080293)
Supplement: Figure S2 — Y-chromosomal phylogenetic chart. Hierarchical phylogenetic relationships and absolute frequencies of the Y-chromosomal haplogroups observed in Libyan and Moroccan populations. Nomenclature is according to Karafet et al. (2008). (PDF) [file pone.0080293.s002.pdf]

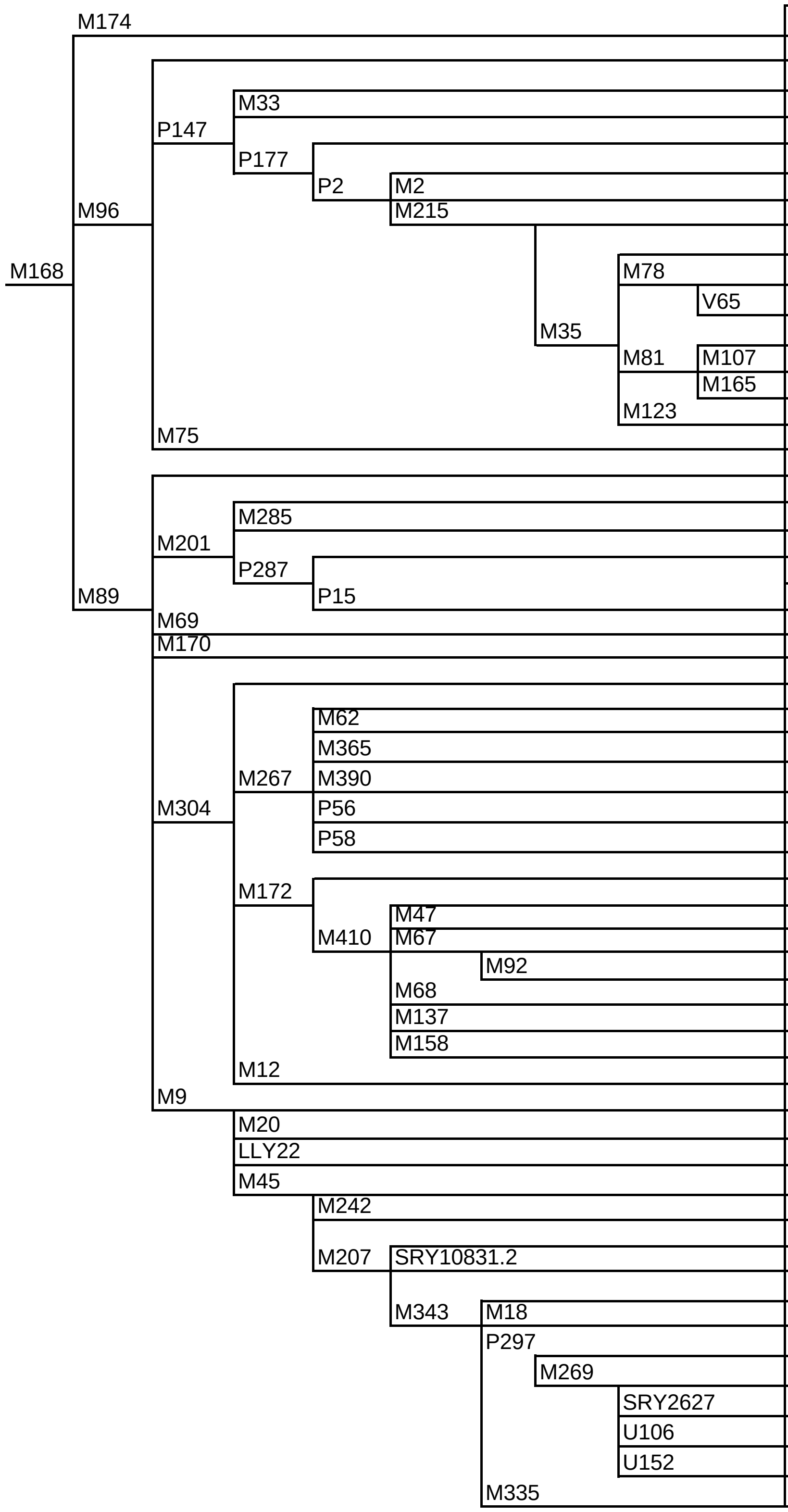

	**Lybians N=215**	**Moroccans N= 82**
D		
E		
E1		
E1a		
E1b		
E1b1		
E1b1a	3	8
E1b1b		
E1b1b1	2	11
E1b1b1a	29	1
E1b1b1a4		4
E1b1b1b	71	5
E1b1b1b1		30
E1b1b1b2		
E1b1b1c	4	
E1b2		
F		
G		
G1		
G2		
G2a	11	
H		
I		
J		
J1		16
J1a		
J1b		
J1c		
J1d		
J1e	80	
J2		7
J2a		
J2a1		
J2a2		
J2a2a	2	
J2a3		
J2a4		
J2a5	7	
J2b		
K		
L		
N		
P		
Q		
R		
R1a	1	
R1b	5	
R1b1a		
R1b1b		
R1b1b2		
R1b1b2d		
R1b1b2g		
R1b1b2h		
R1b1c		
