# Supplementary material for: Genome-Wide and Paternal Diversity Reveal a Recent Origin of Human Populations in North Africa
Source: PLoS One. 2013 Nov 27;8(11):e80293. doi: 10.1371/journal.pone.0080293 (PMC3842387; doi:10.1371/journal.pone.0080293)
Supplement: Table S1 — Populations selected for the Y-chromosome analyses. (DOC) [file pone.0080293.s005.doc]

Table S1: Populations selected for the Y-chromosome analyses.

| Population | Abbreviation | N | References |
| --- | --- | --- | --- |
| **North Africa** |  |  |  |
| Libya | Lib | 215 | Present study |
| Morocco | Mor | 87 | Present study |
| Tunisia | Tun | 239 | Fadhlaoui-Zid et al. 2011; Ennafa et al. 2011 |
| Algeria | Alg | 102 | Robino et al. 2007 |
| Tuareg (Libya) | Tua | 47 | Ottoni et al. 2011 |
| Egypt | Egy | 110 | El-Sibai et al. 2009 |
| **Europe** |  |  |  |
| Italy | Ita | 162 | Onofri et al. 2007 |
| Iberian Peninsula (Andalusians) | And | 168 | Adams et al. 2008 |
| Portugal | Por | 138 | Adams et al. 2008 |
| Basque (Spain) | Bas | 116 | Adams et al. 2008 |
| **Middle East** |  |  |  |
| Lebanon | Leb | 577 | Zalloua et al. 2008 |
| Syria | Syr | 202 | Zalloua et al. 2008 |
| Palestine | Pal | 367 | Zalloua et al. 2008 |
| **Sub-Sahara Africa** |  |  |  |
| Cameroon | Cam | 166 | Montano et al. 2011 |
| Congo | Con | 19 | Montano et al. 2011 |
| Gabon | Gab | 163 | Montano et al. 2011 |
| Nigeria | Nig | 132 | Montano et al. 2011 |
