# Supplementary material for: Genome-Wide and Paternal Diversity Reveal a Recent Origin of Human Populations in North Africa
Source: PLoS One. 2013 Nov 27;8(11):e80293. doi: 10.1371/journal.pone.0080293 (PMC3842387; doi:10.1371/journal.pone.0080293)
Supplement: Table S2 — Y-chromosome haplogroup frequencies in populations selected for the present study. (DOC) [file pone.0080293.s006.doc]

Table S2. Y-chromosome haplogroup frequencies in populations selected for the present study.

|  | **A-M91** | **B-M60** | **E*** | **E-M123** | **E-M2** | **E-M78** | **E-M81** | **G-M201** | **I-M170** | **J-M172** | **J-M267** | **L-M20** | **Q-M242** | **R*** | **R-M17** | **T-M70** |
| --- | --- | --- | --- | --- | --- | --- | --- | --- | --- | --- | --- | --- | --- | --- | --- | --- |
| **Libya** | – | – | 0.0076 | 0.0153 | 0.0878 | 0.1107 | 0.3588 | 0.042 | – | 0.0344 | 0.3053 | – | – | 0.0343 | 0.0038 | – |
| **Morocco** | – | – | – | – | 0.0975 | 0.0610 | 0.5610 | – | – | 0.0854 | 0.1951 | – | – | – | – | – |
| **Algeria** | – | – | – | – | 0.0784 | 0.0588 | 0.4510 | – | – | 0.0490 | 0.2255 | – | 0.0098 | 0.1177 | 0.0098 | – |
| **Tunisia** | – | – | 0.0043 | 0.0043 | 0.0085 | 0.0213 | 0.7906 | 0.0043 | – | 0.0043 | 0.1581 | – | – | – | 0.0043 | – |
| **Tuareg** | – | – | – | – | 0.4255 | – | 0.4894 | – | – | – | – | – | – | 0.0213 | 0.0638 | – |
| **Egypt** | – | – | – | 0.0849 | 0.0377 | 0.3585 | – | 0.0755 | – | 0.0189 | 0.2453 | 0.0189 | – | 0.0849 | 0.0283 | 0.0471 |
| **Lebanon** | – | – | – | 0.0441 | 0.0068 | 0.1085 | 0.0124 | 0.0678 | 0.0497 | 0.2678 | 0.2079 | 0.0542 | 0.0203 | 0.0859 | 0.0260 | 0.0486 |
| **Palestine** | – | – | – | 0.0951 | 0.0035 | 0.1549 | – | 0.0880 | 0.0458 | 0.1972 | 0.3556 | 0.0071 | – | 0.0211 | 0.0106 | 0.0211 |
| **Syria** | – | – | – | 0.0543 | 0.0109 | 0.0815 | 0.0055 | 0.0598 | 0.0489 | 0.2826 | 0.2446 | 0.0543 | – | 0.0544 | 0.0815 | 0.0217 |
| **Andalusia** | – | – | 0.0060 | 0.0060 | – | 0.0238 | 0.0536 | 0.0357 | 0.0595 | 0.1131 | 0.0238 | – | 0.0059 | 0.6488 | 0.0238 | – |
| **Basque** | – | – | – | – | – | – | 0.0086 | – | 0.0776 | 0.0259 | 0.0086 | – | 0.0086 | 0.8707 | – | – |
| **Portugal** | – | – | 0.0362 | 0.0145 | – | – | 0.0580 | 0.1015 | 0.0290 | 0.1160 | 0.0217 | – | – | 0.5145 | 0.0217 | 0.0362 |
| **Italy** | – | – | – | 0.0875 | – | 0.1000 | – | 0.1250 | 0.0812 | 0.1563 | 0.0563 | – | – | 0.3625 | 0.0062 | 0.0250 |
| **Cameroun** | – | 0.0546 | 0.0121 | – | 0.9212 | – | – | – | – | – | – | – | – | 0.0121 | – | – |
| **Congo** | – | 0.0526 | 0.1053 | – | 0.8421 | – | – | – | – | – | – | – | – | – | – | – |
| **Gabon** | – | 0.0797 | 0.0552 | – | 0.8221 | – | – | – | – | – | – | – | – | 0.0430 | – | – |
| **Nigeria** | 0.0303 | 0.0379 | 0.0303 | – | 0.8864 | – | – | – | – | – | – | – | – | 0.0151 | – | – |
