# Supplementary material for: Genome-Wide and Paternal Diversity Reveal a Recent Origin of Human Populations in North Africa
Source: PLoS One. 2013 Nov 27;8(11):e80293. doi: 10.1371/journal.pone.0080293 (PMC3842387; doi:10.1371/journal.pone.0080293)
Supplement: Table S3 — Analyses of Molecular Variance (AMOVA) in North African and Middle Eastern samples based on Y-STR haplotypes and Y-SNP haplogroups. Acronyms are listed in Table S1. (DOC) [file pone.0080293.s007.doc]

Table S3: Analyses of Molecular Variance (AMOVA) in North African and Middle Eastern samples based on Y-STR haplotypes and Y-SNP haplogroups. Acronyms are listed in Table S1.

| **Groups** | **Among groups** | | **Among populations within groups** | | **Within populations** | |
| --- | --- | --- | --- | --- | --- | --- |
|  | **Y-STR** | **Y-SNP** | **Y-STR** | **Y-SNP** | **Y-STR** | **Y-SNP** |
| **All populations** |  |  | 11.15*** | 15.17*** | 88.85*** | 84.83*** |
| **(Mor) vs (Alg, Tun, Tua, Lib , Egy, Leb, Syr, Pal)** | -1.35ns | 0.70ns | 11.41*** | 15.01*** | 89.94*** | 84.30*** |
| **(Mor, Alg) vs (Tun, Tua, Lib, Egy, Leb, Syr, Pal)** | -0.49ns | 2.78ns | 11.29*** | 14.27*** | 89.20*** | 82.95*** |
| **(Mor, Alg, Tun) vs (Tua, Lib, Egy, Leb, Syr, Pal)** | 12.67* | 18.67* | 5.05*** | 5.91*** | 82.29*** | 75.42*** |
| **(Mor, Alg, Tun, Tua) vs (Lib, Egy, Leb, Syr, Pal)** | 10.72* | 19.57** | 5.68*** | 4.91*** | 83.61*** | 75.52*** |
| **(Mor, Alg, Tun, Tua, Lib) vs (Egy, Leb, Syr, Pal)** | 10.78* | 16.63* | 4.60*** | 4.75*** | 84.62*** | 78.62*** |
| **(Mor, Alg, Tun, Tua, Lib, Egy) vs (Leb, Syr, Pal)** | 8.86ns | 12.34ns | 5.52*** | 7.02*** | 85.63*** | 80.64*** |
| **(Mor, Alg, Tun,) vs (Tua, Lib, Egy)** | 0.78ns | 8.33 ns | 3.92*** | 11.43*** | 95.30*** | 80.23*** |
| **(Mor, Alg, Tun,) vs (Tua, Lib, Egy) vs (Leb, Syr, Pal)** | 10.13** | 14.66** | 3.77*** | 4.40*** | 86.10*** | 80.93*** |

*** P<0.0001; ** P<0.01; * P<0.05; ns: not significant
