# Supplementary material for: Genome-Wide and Paternal Diversity Reveal a Recent Origin of Human Populations in North Africa
Source: PLoS One. 2013 Nov 27;8(11):e80293. doi: 10.1371/journal.pone.0080293 (PMC3842387; doi:10.1371/journal.pone.0080293)
Supplement: Table S5 — BATWING results showing times of demographic factors for Y-chromosomes from North Africans. (DOC) [file pone.0080293.s009.doc]

**Table S5.** BATWING results showing times of demographic factors for Y-chromosomes from North Africans

| **Population 1** | **Population 2** | **TMRCA** | **Growth start** | **Split** |
| --- | --- | --- | --- | --- |
| Algerians | Egyptians | 15 (11-25)† | 8.9 (6-12.5) | 2.8 (2.1-3.6) |
| Algerians | Libyans | 15 (10.5-26) | 11 (6.5-18) | 1.3 (0.9-1.9) |
| Algerians | Tuareg | 15.5 (10.5-27) | 7.5 (2.5-14.5) | 1.1 (0.5-2.3) |
| Algerians | Moroccans | 22 (13.5-46) | 1.2 (0.8-2.1) | 1.2 (0.7-1.6) |
| Algerians | Tunisians | 32 (17-74.5) | 1.8 (1.1-2.7) | 1.3 (0.9-1.7) |
| Egyptians | Libyans | 14.5 (10.5-22.5) | 10 (7-14) | 1.9 (1.4-2.5) |
| Egyptians | Tuareg | 16 (11.5-25) | 11 (8-15.5) | 2.8 (1.3-4.6) |
| Egyptians | Moroccans | 15 (10.5-24) | 9.5 (6.7-13.2) | 2.7 (2-3.6) |
| Egyptians | Tunisians | 15 (11-32) | 10 (7.3-13.5) | 2.6 (2-3.4) |
| Libyans | Tuareg | 16.5 (11-27) | 13 (7.5-22.5) | 1.9 (1.1-3.2) |
| Libyans | Moroccans | 14.5 (10-25.5) | 12 (3.7-19.5) | 1.3 (0.8-1.9) |
| Libyans | Tunisians | 30.5 (14-79) | 10.7 (0.6-24) | 1 (0.7-1.4) |
| Moroccans | Tunisians | 42 (20.5-96) | 1.4 (0.6-7.5) | 1.2 (0.8-1.8) |
| Tuareg | Moroccans | 15 (10-26) | 8.5 (2.3-18) | 1.6 (0.7-3.2) |
| Tuareg | Tunisians | 44.5 (21-98.5) | 1.2 (0.2-25.5) | 1.7 (1-2.9) |

† Median value with 95%CI in thousand of years ago
