# Supplementary material for: Genome-Wide and Paternal Diversity Reveal a Recent Origin of Human Populations in North Africa
Source: PLoS One. 2013 Nov 27;8(11):e80293. doi: 10.1371/journal.pone.0080293 (PMC3842387; doi:10.1371/journal.pone.0080293)
Supplement: Table S6 — 3-population test showing gene flow to North Africans. (DOC) [file pone.0080293.s010.doc]

| **Target** | **Source1** | **Source2** | **Minimum^a^ ƒ3** | **S.E.** | **Z-score** |
| --- | --- | --- | --- | --- | --- |
| Algerians | Yoruba | Basque | -0.00856386^b^ | 0.000216115 | -39.6263 |
| Algerians | Yoruba | Lebanese Christians | -0.00712749 | 0.000181691 | -39.2285 |
| Egyptians | Yoruba | Basque | -0.0064804 | 0.00020486 | -31.6333 |
| Egyptians | Yoruba | Lebanese Christians | -0.00573643 | 0.000178883 | -32.0681 |
| Libyans | Yoruba | Basque | -0.00777087 | 0.00019491 | -39.8689 |
| Libyans | Yoruba | Lebanese Christians | -0.00664719 | 0.000161584 | -41.1377 |
| North Moroccans | Yoruba | Basque | -0.00657986 | 0.00019616 | -33.5433 |
| North Moroccans | Yoruba | Lebanese Christians | -0.00475158 | 0.000160078 | -29.683 |
| South Moroccans | Yoruba | Basque | -0.0111038 | 0.000202255 | -54.9001 |
| South Moroccans | Yoruba | Lebanese Christians | -0.00977224 | 0.000175436 | -55.7025 |
| Saharawi | Yoruba | Basque | -0.00677832 | 0.000228239 | -29.6983 |
| Saharawi | Yoruba | Lebanese Christians | -0.00557365 | 0.000200604 | -27.7843 |
| Tunisian Berbers | Yoruba | Basque | 0.000352494^c^ | 0.000275206 | 1.28084 |
| Tunisian Berbers | Yoruba | Lebanese Christians | 0.00209835 | 0.000252313 | 8.31648 |

**Table S6.** 3-population test showing gene flow to North Africans

^a^ The table only lists the two lowest f3 statistics observed in North Africans.

^b^ A significantly negative value of the f3 statistic implies that target population is admixed.

^c^ A positive f3 suggests that target population is unadmixed.
